# Clinics Given at the Tri-State Dental Meeting

**Published:** 1901-09-15

**Authors:** 


### CLINICS GIVEN AT THE TRI-STATE DENTAL MEETING.

### PORCELAIN INLAYS.

BY DR. E. E. REESE, INDIANAPOLIS, IND.

An impression of the cavity is taken in oxy-phosphate cement, in which has been incorporated a little soap-stone. Soap-stone is dusted in the cavity also, before the cement is applied, to facilitate the drawing of the cast after it has hardened. The cast is reinforced with cement and imbedded in plaster. The matrix material is then swaged over the cement cast and imbedded in investment material. A porcelain tooth of the correct shade is then selected, pulverized and used as the body for the inlay, and fused into the matrix. The inlay is then set and finished in the usual manner.

### A METHOD OF MOUNTING AND TRUING WHEELS FOR ENGINE AND LATHE.

BY DR. GEORGE B. PERRY, CHICAGO, ILLS.

Truing carborundum wheels by means of a diamond is effectively accompished by the method of the clinician, which is as follows: Select a screw-top mandrel such as is suitable for the stone, place melted sealing wax on thread of the screw, pass screw end through wheel and into the shank. The sealing wax is used to hold the screw tightly in the shank, so that no matter which way the lathe is turned the screw can not loosen.

Get the wheel as true as possible before the wax hardens, then cool and fix in water. The mandrel, with wheel attached, is now placed in the lathe and the wheel is further trued by means of a diamond set in a steel shank.

---

### REMOVAL OF EXCESS CEMENT IN SETTING CROWNS.

---

BY DR. S. D. RUGGLES, PORTSMOUTH, O.

---

After the crown is ready for setting, take a wooden toothpick with a tuft of cotton wound around one end, and smear the cotton with a little vaseline. Draw the vaselined cotton around the outside of the band fitted to the crown, and proceed to set the crown in the ordinary way with cement. The vaseline prevents the cement sticking to the band, so that any surplus may be lifted off with an explorer or other fine-pointed instrument. In this way all of the surplus cement is gotten rid of and no particles are left to irritate the gum margin and cause inflammation, etc.

---

### COMBINATION FILLING, AMALGAM AND GOLD, AT THE SAME SITTING.

---

BY DR. J. W. CLARK, LOUISVILLE, KY.

---

The operation consisted of filling the cavity about two-thirds full of amalgam, filling over this with Delrey's gold, which, uniting with the amalgam, formed a perfect union and solid filling. A matrix was employed and held tightly in place on the tooth by means of soaped ligatures.

### ROOT DRIER.

BY DR. H. P. CARLETON, SAN FRANCISCO, CAL.

This invention consists of platinum wire on asbestos fiber enclosed in a glass tube, and fastened in plaster. The air is thrown through the tube over the heated platinum and regulated as to temperature by the amount of air used.

